# ROP16 Promotes Epithelial‐Mesenchymal Transition‐Like Changes in Ocular Toxoplasmosis via STAT3 and TGF‐β1 Pathways

**DOI:** 10.1155/tbed/9126072

**Published:** 2026-05-22

**Authors:** Lingling Song, Lihui Xu, Yao Liu, Yun Yang, Cong Wang, Ya Zhang, Qingli Luo, Yuanyuan Cao, Yong Wang, Li Yu

**Affiliations:** ^1^ Department of Microbiology and Parasitology, Anhui Provincial Key Laboratory of Zoonoses, School of Basic Medical Sciences, Anhui Medical University, Hefei, Anhui, China, ahmu.edu.cn; ^2^ Department of Clinical Laboratory, The People’s Hospital of Lujiang, Lujiang, Anhui, China; ^3^ Department of Clinical Laboratory, The Second People’s Hospital of Hefei, Hefei, Anhui, China, ahmu.edu.cn; ^4^ Department of Ophthalmology, The First Affiliated Hospital of Anhui Medical University, Hefei, Anhui, China, ahmu.edu.cn; ^5^ Department of Clinical Laboratory, The First Affiliated Hospital of Anhui Medical University, Hefei, Anhui, China, ahmu.edu.cn

**Keywords:** EMT-like changes, OT, ROP16, STAT3, TGF-β1, *Toxoplasma gondii*

## Abstract

Toxoplasmosis is a globally significant infectious disease, affecting approximately one‐third of the world’s population. Ocular toxoplasmosis (OT) is a major and vision‐threatening clinical manifestation. However, its pathogenesis remains elusive, and effective targeted therapies are unavailable. This study demonstrates that *Toxoplasma gondii* infection induces epithelial‐mesenchymal transition (EMT)‐like changes in both human retinal pigment epithelial (RPE) cells (ARPE‐19) and murine ocular tissues. Mechanistic investigations, employing rhoptry protein 16 (ROP16) knockout parasites and ROP16 overexpression models, revealed that the parasite‐derived protein ROP16 promotes EMT‐like changes by activating the host signal transducer and activator of transcription 3 (STAT3) and transforming growth factor β1 (TGF‐β1) signaling pathway. Furthermore, pharmacological inhibition of STAT3 or TGF‐β1 significantly attenuated EMT‐like changes in vitro and ameliorated OT pathology in mice. In summary, our findings identify the ROP16‐STAT3 and TGF‐β1 pathways as a key regulatory pathway in OT pathogenesis, providing novel insights into the disease mechanism and suggesting STAT3 and TGF‐β1 inhibitors as promising therapeutic candidates.

## 1. Introduction

*Toxoplasma gondii* (*T. gondii*) is an obligate intracellular parasitic protozoan with a global distribution. Epidemiological studies have shown that about one‐third of the world’s population is infected with *T. gondii*, although there are significant geographical differences in serum prevalence. Africa had the highest infection rate (61.4%), followed by Oceania (38.5%), South America (31.2%), and Europe (29.7%), while North America (17.5%) and Asia (16.4%) had the lowest infection rate. These differences were mainly attributed to dietary habits, public health conditions, and dominant *T. gondii* genotypes [1, 2]. Based on molecular markers and virulence phenotypes of mouse models, *T. gondii* can be classified into three typical genotypes (types I, II, and III) and atypical variations [3, 4]. Unlike the dominant strains in North America and Europe, which are type II, the *T. gondii* population in China has a unique genetic structure and significant genetic diversity. Chinese genotype 1, represented by WH3 and WH6, dominated the population structure [5, 6].

Among human cases of toxoplasmosis, ocular toxoplasmosis (OT) is the second most common clinical manifestation after cerebral toxoplasmosis. The main clinical manifestations of OT are structural damage, severe inflammation, and irreversible visual impairment. In immunocompetent hosts, active infections usually resolve spontaneously within 2–4 months. The repair process is marked by the centripetal regression of inflammatory lesions, which eventually leads to atrophic changes, followed by the formation of pigmented chorioretinal scars due to the irreversible destruction of the retinal pigment epithelium (RPE) [7, 8]. In Europe, ~3 million people suffer from retinopathy or retinal scarring [9]. Long‐term research conducted by the research team shows that, based on the definition of recurrence and the length of follow‐up time, ~60%–86% of patients experience recurrence [10, 11]. In view of its high blindness rate and recurrence rate, as well as irreversible retinal structural damage and severe visual impairment, it is of great clinical and public health significance to study its pathogenesis and formulate effective prevention and treatment strategies [11]. A central pathological feature of these irreversible lesions is fibrosis, a process driven largely by epithelial‐mesenchymal transition (EMT) [12].

The transforming growth factor β1 (TGF‐β1) pathway is one of the most effective mechanisms for inducing EMT. This ligand binds to the type II and type I receptor complexes on the cell surface, activating receptor kinases and leading to specific phosphorylation of Smad family member 2 (SMAD2) and Smad family member 3 (SMAD3). Phosphorylated SMAD2/3 forms a trimer with SMAD4 and translocates into the nucleus. This process usually upregulates the core EMT transcription factor and inhibits the expression of epithelial markers such as E‐cadherin, thus effectively starting the EMT process [13]. On the other hand, inflammatory cytokines such as interleukin‐6 (IL‐6) strongly induce EMT by binding to receptors, activating Janus kinase (JAK), and leading to phosphorylation of signal transducer and activator of transcription 3 (STAT3) [14]. Phosphorylated STAT3 (pSTAT3) forms a dimer, enters the nucleus, and directly binds to the promoter of the EMT transcription factor, enhancing its transcription [15]. Studies have shown that TGF‐β1 can induce the production of IL‐6 under certain circumstances, thus activating the JAK‐STAT3 pathway, forming a positive feedback loop and synergistically enhancing EMT [16]. STAT3 can also physically interact with the SMAD complex or jointly regulate a group of overlapping downstream target genes, thereby achieving precise and synergistic regulation of EMT at the transcriptional level [17]. In view of the central role of these two pathways in EMT, small‐molecule inhibitors targeting TGF‐β1 receptor kinase and the JAK‐STAT3 pathway have become important strategies to block the progress of EMT and inhibit fibrotic diseases and tumor metastasis [18–21]. Given the efficacy of these inhibitors in other fibrotic contexts, we sought to investigate their potential relevance in ocular fibrotic diseases, particularly those involving the RPE.

In ocular tissues, EMT is a core pathological mechanism driving various proliferative and fibrotic diseases [22–25]. Notably, RPE cells are regarded as a primary source of myofibroblasts in retinal fibrosis, with EMT being the central mechanism through which RPE cells contribute to fibrosis [26, 27]. When retinal damage occurs, RPE cells are activated, lose epithelial features, and acquire mesenchymal markers, transforming into myofibroblasts that migrate and proliferate to maintain retinal integrity. However, when the damage is excessive or the microenvironment is persistently stimulated by inflammatory cytokines, the reparative response of RPE cells becomes dysregulated, ultimately leading to pathological fibrosis. In OT, the chronic inflammatory environment and direct interactions between the parasite and host cells may trigger EMT‐like changes in RPE cells, thereby exacerbating retinal structural destruction and scar formation. However, the specific mechanisms underlying EMT‐like changes induction in OT remain unclear. Thus, investigating whether OT is mediated by EMT‐like changes and elucidating the underlying mechanisms hold significant scientific value. Addressing this question is crucial for deepening the understanding of OT’s pathogenesis and developing interventional strategies targeting the fibrotic process.

When *T. gondii* invades host cells, it secretes numerous effector proteins to modulate host signaling pathways and immune responses, facilitating its own persistent infection [28]. Among them, rhoptry protein 16 (ROP16) is a key effector protein identified through forward genetic hybridization technology. It is considered to be a key determinant of the diversity of *T. gondii* virulence and has genetic polymorphism characteristics [29, 30]. Previous studies have confirmed that ROP16 is a kinase that phosphorylates STAT3 and STAT6, thereby regulating host biological activities [31]. Activation of STAT3 by various stimuli is a well‐established mechanism driving EMT in cancer and fibrosis. Given that ROP16 is a potent activator of STAT3, we therefore hypothesize that during ocular *T*. *gondii* infection, the parasite effector ROP16 acts as a key trigger for EMT‐like changes by hijacking the host STAT3 signaling pathway. This hypothesis links the parasite‐specific pathogenic strategies with the host cell processes that drive pathological tissue remodeling.

## 2. Materials and Methods

### 2.1. Parasites, Cell Culture, and Treatments

The *T. gondii* strains used in the present study, including WH3 (wild‐type), WH3Δ*rop16*, and WH6, were obtained from and are maintained by the Anhui Provincial Key Laboratory of Zoonoses. These strains were propagated in African green monkey kidney (Vero) cells (purchased from ATCC) and cultured in Dulbecco’s modified Eagle medium (DMEM) (Gibco) supplemented with 10% fetal bovine serum (FBS) (Hyclone). ARPE‐19 cells were cultured in DMEM/F‐12 medium (Gibco) supplemented with 10% FBS, penicillin (100 U/mL), and streptomycin (100 μg/mL) in a humidified 5% CO_2_ atmosphere at 37°C. After the cells achieved 80% confluence, they were passaged at a ratio of 1:3. S3I‐201 (Selleck Chemicals) and TGF‐β1‐IN‐1 (Med Chem Express) were individually added to the culture medium at specified concentrations 6 h prior to *T. gondii* infection. The concentrations of S3I‐201 (100 µM) and TGF‐β1‐IN‐1 (5 µM) were chosen according to previously published studies, where these concentrations demonstrated effective inhibition of the target pathways while exhibiting minimal cytotoxic effects [32, 33].

### 2.2. In Vivo Mouse Experiments

Acute infection model: Female C57BL/6 mice aged 8–10 weeks were housed under specific pathogen‐free (SPF) conditions with free access to standard laboratory chow and tap water, maintained on a 12‐h light/dark cycle. All experimental procedures involving animals were conducted in accordance with the National Guidelines for Animal Use in Research (China), and permission was obtained from the Institute of Health and Medicine, Hefei Comprehensive National Science Center (IHM‐AP‐2025‐162). Mice were randomly assigned to experimental groups: WH3 infection (*n* = 5), WH3Δ*rop16* infection (*n* = 5), WH3 infection + STAT3 inhibitor (S3I‐201) treatment (*n* = 5), and WH3 infection + TGF‐β receptor inhibitor (TGF‐β1‐IN‐1) (*n* = 5) treatment. Freshly egressed tachyzoites were resuspended in PBS at a concentration of 5 × 10^5^ parasites/10 µL. Mice were anesthetized by inhalation of 4% isoflurane for 2–5 min, with the adequacy of anesthesia confirmed by the loss of consciousness and absence of response to noxious stimuli. Then, 10 µL of the parasite suspension (or PBS for controls) was injected intravitreally. Treatment groups received intravitreal injections of S3I‐201 (10 mg/kg) or TGF‐β1‐IN‐1 (30 mg/kg) on days 2, 4, 6, 8, 10, 12, and 14 postinfection [34, 35]. On day 15, mice were anesthetized by inhalation of 4% isoflurane for 2–5 min. While under deep anesthesia, they were immediately euthanized by cervical dislocation. Then, eyeballs were enucleated for subsequent analysis.

Chronic infection model (bradyzoites/cysts): Mice were randomly divided into the following groups: WH6 cyst infection (*n* = 5), WH6 cyst infection + S3I‐201 treatment (*n* = 5), and WH6 cyst infection + TGF‐β1‐IN‐1 treatment (*n* = 5). Mice were orally gavaged with 20 freshly isolated tissue cysts per mouse. Treatment groups received a daily intraperitoneal injection of S3I‐201 (10 mg/kg) or TGF‐β1‐IN‐1 (30 mg/kg). On day 30 postinfection, animals were deeply anesthetized by inhalation of 4% isoflurane for 2–5 min and, while under deep anesthesia, were immediately euthanized by cervical dislocation. Then, eyeballs were harvested for analysis.

Humane endpoints and animal monitoring: Explicit humane endpoints were predefined to minimize suffering. Animals were monitored throughout the study and would have been euthanized immediately if meeting any endpoint: >20% body weight loss, severe lethargy/mobility issues, inability to eat/drink, labored breathing, hunched posture, ruffled fur, or unresponsiveness. No animals met these criteria; all were processed at the scheduled endpoints (day 15 for acute and day 30 for chronic models).

### 2.3. Hematoxylin and Eosin (H&E), Masson Staining, and Immunohistochemistry

H&E staining: Fresh tissue samples were fixed in 4% paraformaldehyde for over 24 h, dehydrated through a graded ethanol series, cleared in xylene, and embedded in paraffin. Sections (4 µm thick) were cut, mounted on slides, and dried. Sections were deparaffinized in xylene and rehydrated through a graded ethanol series to water. They were then stained with hematoxylin, differentiated, blued, counterstained with eosin, dehydrated, cleared, and mounted with neutral balsam. Stained sections were examined and imaged under a light microscope.

Masson staining: Paraffin‐embedded tissue sections were deparaffinized and rehydrated as described for H&E staining. Sections were mordanted in Bouin’s fluid overnight. After washing, nuclei were stained with Weigert’s iron hematoxylin. Cytoplasm and muscle fibers were stained with Biebrich scarlet‐acid fuchsin solution. Collagen fibers were differentially stained with aniline blue after treatment with phosphomolybdic/phosphotungstic acid. Sections were dehydrated, cleared, and mounted. Collagen appears blue, muscle fibers red, and nuclei blue‐black under microscopy.

Immunohistochemical (IHC) staining: Eye tissue sections (5 µm) were incubated with primary antibodies against EMT‐related markers at 4°C overnight, followed by incubation with horseradish peroxidase (HRP)‐conjugated secondary antibodies for 1 h at room temperature. The 3,3^′^‐diaminobenzidine (DAB) substrate was applied to develop brown reaction products. Images were captured using a light microscope.

### 2.4. Oligonucleotide and Plasmid Transfection

siRNA oligonucleotides were commercially obtained from Sangon Biotech. The sequences of si‐STAT3 (1), si‐STAT3 (509), and si‐STAT3 (538) are listed in Supporting Information 1: Table S1. Cells were seeded into six‐well plates, allowed to reach 80%–90% confluence, and then transfected with si‐NC or si‐STAT3 using Lipofectamine 3000 (Invitrogen) for 24 h. For plasmid transfection, ARPE‐19 cells were seeded and allowed to reach 50%–60% confluency. Plasmids containing WT ROP16 or ROP16 (L503S) were introduced into the cells using Lipofectamine 3000 according to the manufacturer’s instructions (Invitrogen).

### 2.5. Scratch Assay

ARPE‐19 cells were seeded in six‐well plates and grown to confluence. A straight scratch was made on the cell monolayer using a sterile pipette tip. After washing to remove debris, fresh medium was added. Images were captured at 0 and 24 h postscratching. The migration area was quantified using ImageJ software and expressed as the percentage of wound closure relative to the initial scratch area.

### 2.6. Transwell Migration Assay

Infected or treated cells were harvested by trypsinization, neutralized with serum‐free medium, and centrifuged. The cell pellet was resuspended in serum‐free medium at a density of 1 × 10^5^ cells/mL. The lower chamber of a 24‐well transwell plate (Corning) was filled with 600 µL of medium containing 10% FBS as a chemoattractant. The cell suspension (200 µL) was added to the upper chamber. After 24 h of incubation, nonmigratory cells on the upper surface of the membrane were removed with a cotton swab. Cells that had migrated to the lower surface were fixed with 4% PFA, stained with 0.1% crystal violet, and imaged. The number of migrating cells was counted from five random fields under a microscope, and quantitative analysis was performed using ImageJ software.

### 2.7. Quantitative Real‐Time PCR (qPCR)

Total RNA was extracted from samples using a commercial kit. RNA was reverse‐transcribed into cDNA. The cDNA was diluted to a working concentration of 10 ng/µL. qPCR was performed using a SYBR Green mix. Each 10 µL reaction contained 5 µL of 2× SYBR Green mix, 0.3 µL each of forward and reverse primers (10 µM), 2.4 µL nuclease‐free water, and 2 µL of template. Primers are listed in Supporting Information 1: Table S1. Reactions were run on a LightCycler 96 system. Relative gene expression was calculated using the 2^(–ΔΔCt)^ method.

### 2.8. Immunofluorescence Assay (IFA)

Cells grown on coverslips were infected with freshly egressed tachyzoites for 24 h. Cells were washed with PBS, fixed with 4% paraformaldehyde for 30 min, and permeabilized with 0.1% Triton X‐100 for 5 min. After blocking with 3% BSA for 1 h at 37°C, cells were incubated with primary antibody diluted in 1% BSA overnight at 4°C. Following PBS washes, cells were incubated with fluorophore‐conjugated secondary antibody (in 1% BSA) for 1 h at 37°C in the dark. Nuclei were counterstained with DAPI. Coverslips were mounted onto glass slides using antifade mounting medium and imaged using a confocal microscope.

### 2.9. Protein Extraction and Western Blot Analysis

Samples were lysed on ice for 30 min using RIPA lysis buffer supplemented with 1% PMSF protease inhibitor (Beyotime Biotechnology), with vortex mixing every 10 min. The lysate was centrifuged at 12,000 × *g* for 15 min at 4°C. The supernatant was collected, and protein concentration was determined using a BCA assay. Samples were normalized, mixed with 5× SDS‐PAGE loading buffer (Beyotime Biotechnology) at a 4:1 ratio, and denatured at 100°C for 10 min. Proteins were separated by SDS–PAGE using a 10% gel, alongside a prestained protein molecular weight marker (Thermo Fisher Scientific, Catalog # 26616), and transferred onto a PVDF membrane (Merck Millipore). The membrane was blocked with 5% nonfat milk or BSA in TBST for 2 h at room temperature and then incubated with primary antibody overnight at 4°C. After washing, the membrane was incubated with an HRP‐conjugated secondary antibody for 1 h. Protein bands were visualized using an enhanced chemiluminescence (ECL) substrate and imaged.

### 2.10. Statistical Analysis

Statistical analyses were performed using GraphPad Prism software. Comparisons between two groups were assessed using a two‐tailed unpaired *t*‑test. For comparisons involving more than two groups, one‑way analysis of variance (ANOVA) was used. For experiments comparing treated cells of different genotypes, two‑way ANOVA was applied. In all figures, error bars represent the mean ± standard error of the mean (SEM). Sample sizes and the number of independent experiments are provided in the corresponding figure legends. Statistical significance was set at  ^∗^ *p* < 0.05,  ^∗∗^ *p* < 0.01,  ^∗∗∗^ *p* < 0.001, and  ^∗∗∗∗^ *p* < 0.0001.

## 3. Results

### 3.1. WH3 Infection Induces EMT‐Like Changes in Human RPE Cells

**Figure 1:**
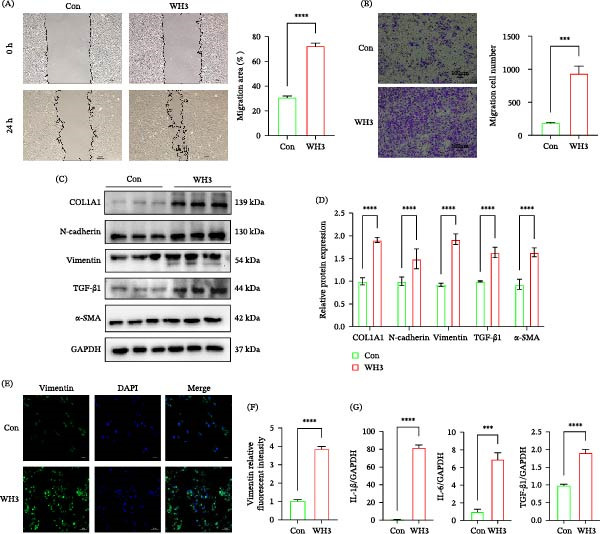
WH3 infection induces EMT‐like changes in ARPE‐19 cells. (A) Representative images of ARPE‐19 cells infected with WH3 at 0 and 24 h after scratching (*n* = 3 per group). Scale bar: 100 μm. The relative scratch area was measured using ImageJ software. (B) Representative images of the transwell assay in ARPE‐19 cells with or without WH3 infection (*n* = 3 per group). The number of migrating cells was evaluated based on three random fields under a microscope, and quantitative analysis was performed using ImageJ software. Scale bar: 100 μm. (C, D) Protein expression and quantification of EMT‐related markers in ARPE‐19 cells with or without WH3 infection (*n* = 3 per group). (E, F) Representative confocal images of vimentin (green) in ARPE‐19 cells with or without WH3 infection (*n* = 3 per group). Quantitative analysis of vimentin fluorescence intensity. (G) qPCR detection of cytokines in ARPE‐19 cells with or without WH3 infection (*n* = 3 per group). Data are presented as mean ± SEM.  ^∗^ *p* < 0.05,  ^∗∗^ *p* < 0.01,  ^∗∗∗^ *p* < 0.001, and  ^∗∗∗∗^ *p* < 0.0001.

To investigate whether WH3 infection induces EMT‐like changes in ARPE‐19 cells, a series of experiments were performed. The wound healing assay demonstrated that compared with the uninfected control group, WH3 infection significantly enhanced the migratory capacity of ARPE‐19 cells, as evidenced by a marked reduction in wound area (Figure 1A). The transwell migration assay further revealed that WH3 infection substantially increased the number of cells migrating through the membrane, confirming the promigratory phenotype induced by *T. gondii* (Figure 1B). Western blot analysis and quantitative assessment of EMT marker expression in infected versus control in ARPE‐19 cells showed that WH3 infection led to significant upregulation of mesenchymal markers (Figure 1C,D), indicating a characteristic shift in the EMT protein expression profile. Immunofluorescence analysis also confirmed that WH3 infection markedly upregulated the expression of vimentin in ARPE‐19 cells (Figure 1E,F). qPCR analysis of profibrotic cytokine mRNA expression levels demonstrated that WH3 infection significantly upregulated the transcription of profibrotic cytokines (Figure 1G). These results show that WH3 infection promotes EMT‐like changes in ARPE‐19 cells by activating EMT‐related signaling.

### 3.2. Both Acute and Chronic *T. gondii* Infections Can Effectively Induce EMT‐Like Changes in Ocular Tissues

**Figure 2:**
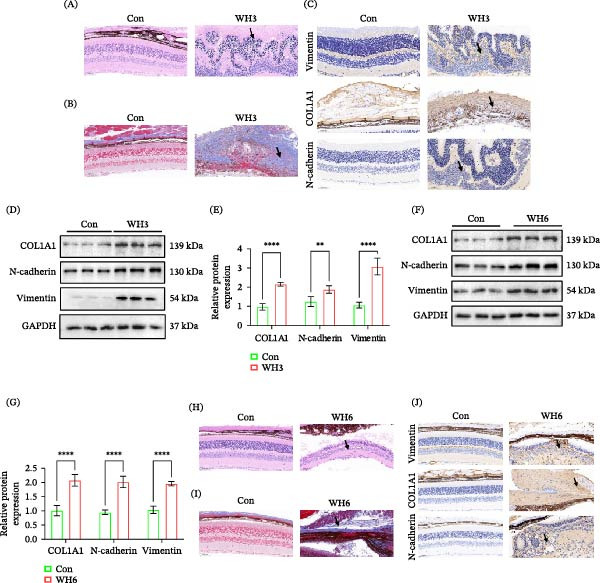
Acute and chronic *T. gondii* infections potently induce ocular EMT‐like changes. (A, B) Histopathological features of ocular tissues from C57BL/6 mice following intravitreal injection of PBS or the WH3 strain, as shown by H&E (A) and Masson staining (B). (C) Expression and distribution of vimentin, COL1A1, and N‐cadherin detected by immunohistochemical (IHC) staining in ocular tissues from C57BL/6 mice intravitreally injected with PBS or WH3. (D, E) Protein expression and quantitative analysis of EMT‐related markers in ocular tissues from C57BL/6 mice treated with PBS or WH3 (*n* = 5 per group). (F, G) Protein expression and quantitative analysis of EMT‐related markers in ocular tissues from C57BL/6 mice treated with PBS or WH6. (H, I) Histopathological features of ocular tissues from C57BL/6 mice treated with PBS or WH6 cysts, as shown by H&E (H) and Masson staining (I). (J) Expression and distribution of vimentin, COL1A1, and N‐cadherin detected by IHC staining in ocular tissues from C57BL/6 mice following oral gavage with PBS or WH6 cysts (*n* = 5 per group). Arrows in subparts (A, B, C, H, I, and J) indicate areas of pathological damage, collagen deposition, or positively stained lesions. Data are presented as mean ± SEM.  ^∗^ *p* < 0.05,  ^∗∗^ *p* < 0.01,  ^∗∗∗^ *p* < 0.001, and  ^∗∗∗∗^ *p* < 0.0001.

To investigate the effects of *T. gondii* infection on ocular histopathological alterations and EMT‐like changes, this study employed C57BL/6 mice infected with both acute hypervirulent (WH3) and chronic hypovirulent (WH6) strains. For the acute infection model, WH3 was injected into the vitreous cavity of mice. On day 15 postinfection, ocular tissues were harvested for pathological examination. Results revealed that compared to the PBS control group, H&E staining of ocular tissues from WH3‐infected mice demonstrated severe disruption of normal retinal layering accompanied by extensive inflammatory cell infiltration (Figure 2A). Masson staining further showed that there was obvious deposition of blue collagen fibers in the infected eyes, indicating the beginning of tissue fibrosis (Figure 2B). IHC staining showed that the expression levels of vimentin, COL1A1, and N‐cadherin in the eye tissues infected with WH3 were significantly higher than those in the control group (Figure 2C). These proteins were extensively distributed within the lesion areas, consistent with the spatial distribution of tissue fibrosis and mesenchymal cell transformation. Compared with the control group, acute WH3 infection also upregulated the expression of EMT‐related proteins in ocular tissues (Figure 2D,E), indicating that acute infection strongly activated the EMT‐like changes process in ocular tissues. Meanwhile, the chronic infection model was established by intragastric administration of WH6 cysts. Western blot analysis indicated that chronic WH6 infection significantly increased the expression of vimentin, COL1A1, and N‐cadherin in the eyes (Figure 2F,G). H&E staining revealed that even the less virulent WH6 chronic infection could cause significant structural damage to ocular tissues, including retinal disorders and inflammatory infiltration (Figure 2H). Consistent with the results of WH3 infection, Masson staining showed that chronic WH6 infection also induced significant collagen deposition, which was further confirmed by the upregulated expression of EMT‐related proteins in immunohistochemistry (Figure 2I,J). This confirms that both acute and chronic *T. gondii* infection are effective inducers of EMT‐like changes in the eye.

### 3.3. *T. gondii* Infection Induces EMT‐Like Changes in ARPE‐19 Cells via Activation of the STAT3 and TGF‐β1 Signaling Pathway

Our results showed that *T. gondii* infection not only induced EMT‐like changes but also was accompanied by significant upregulation of p‐STAT3 (Figure 3A). This finding suggests that the STAT3 signaling pathway may be involved in the regulation of EMT‐like changes associated with WH3 infection. To test this hypothesis, we first pretreated cells with STAT3 inhibitor S3I‐201, then infected cells with WH3, and analyzed phenotypes related to EMT‐like changes. Wound‐healing assays demonstrated enhanced migration capacity in infected cells, while S3I‐201 treatment significantly suppressed this effect (Figure 3B,C). Transwell assays further confirmed increased transmembrane cell migration postinfection, which was reversed by S3I‐201 (Figure 3D,E). We further knocked down STAT3 expression using small interfering RNA (siRNA), and the knockdown efficiency was validated by Western blot analysis (Figure 3F). Results showed that siRNA‐mediated knockdown significantly reduced EMT marker expression levels compared to the infection group, consistent with the inhibitor experiment findings (Figure 3G,H). Additionally, Western blot analysis revealed that STAT3 inhibitor treatment also reversed WH3‐induced changes in EMT marker expression (Figure 3I,J). Furthermore, the TGF‐β1 pathway, another classic inducer of EMT‐like changes, exhibits crosstalk with STAT3 signaling. Our data indicate that *T. gondii* infection also upregulates TGF‐β1 expression. To further investigate the role of TGF‐β1 in infection‐induced EMT‐like changes, we intervened using its specific inhibitor, TGF‐β1‐IN‐1. Similar to the STAT3 inhibition experiment, TGF‐β1‐IN‐1 pretreatment significantly suppressed WH3 infection‐promoted cell migration and invasion (Figure 3K–N). At the molecular level, this treatment similarly reversed changes in the expression of EMT‐associated proteins (Figure 3O,P). Immunofluorescence analysis confirmed that WH3 infection markedly upregulated vimentin expression in ARPE‐19 cells. However, this upregulation was abolished by treatment with either S3I‐201 or TGF‐β1‐IN‐1 (Figure 3Q,R). Overall, these results suggest that WH3 infection may synergistically induce EMT‐like changes by simultaneously activating the STAT3 and TGF‐β1 signaling pathways. Inhibiting either of these pathways alone can reverse the EMT‐like changes in phenotype caused by WH3.

Figure 3
*T. gondii* infection induces EMT‐like changes in ARPE‐19 cells via activation of the STAT3 and TGF‐β1 signaling pathways. (A) Protein expression and quantification of p‐STAT3 and p‐SMAD in ARPE‐19 cells with or without WH3 infection (*n* = 3 per group). (B, C) Representative images and quantitative analysis of ARPE‐19 cells under the following conditions: control, WH3 infection, and WH3 infection with S3I‐201 treatment, captured at 0 and 24 h postscratching (*n* = 3 per group). The relative wound area was measured using ImageJ software. Scale bar: 100 μm. (D, E) Representative images and quantification of cell migration in ARPE‐19 cells treated as indicated: control, WH3 infection, and WH3 infection with S3I‐201 (*n* = 3 per group). Scale bar: 100 μm. Migrating cells were counted from three random microscopic fields and analyzed using Image J. (F) Western blot analysis and quantification of the efficiency of STAT3 knockdown by siRNA in ARPE‐19 cells (*n* = 3 per group). (G, H) Protein expression and quantification of EMT‐related markers in ARPE‐19 cells transfected with control siRNA or STAT3‐targeting siRNA, followed by WH3 infection (*n* = 3 per group). (I, J) Western blot analysis and quantitative analysis of EMT‐related markers in ARPE‐19 cells under the following conditions: control, WH3 infection, and WH3 infection with S3I‐201 (*n* = 3 per group). (K, L) Scratch wound healing assay with TGF‐β1 inhibition. Representative images and quantification of ARPE‐19 cells under the following conditions: PBS control, WH3 infection, and WH3 infection with TGFβ‐IN‐1 treatment (*n* = 3 per group). (M, N) Transwell migration assay with TGF‐β1 inhibition. Representative images and quantification of cell migration in ARPE‐19 cells treated with PBS control, WH3 infection, or WH3 infection with TGFβ‐IN‐1 (*n* = 3 per group). (O, P) Protein expression and quantification of EMT‐related markers in ARPE‐19 cells treated with control, WH3 infection, or WH3 infection with TGFβ‐IN‐1 (*n* = 3 per group). (Q, R) Vimentin cytoskeleton analysis. Representative confocal images (Q) and quantitative analysis (R) of vimentin (green) in ARPE‐19 cells across four conditions: PBS control, WH3 infection, WH3 infection with S3I‐201, and WH3 infection with TGF‐β1‐IN‐1 (*n* = 3 per group). Fluorescence intensity was quantified using ImageJ. Data are presented as mean ± SEM.  ^∗^ *p* < 0.05,  ^∗∗^ *p* < 0.01,  ^∗∗∗^ *p* < 0.001, and  ^∗∗∗∗^ *p* < 0.0001.

**Figure:**
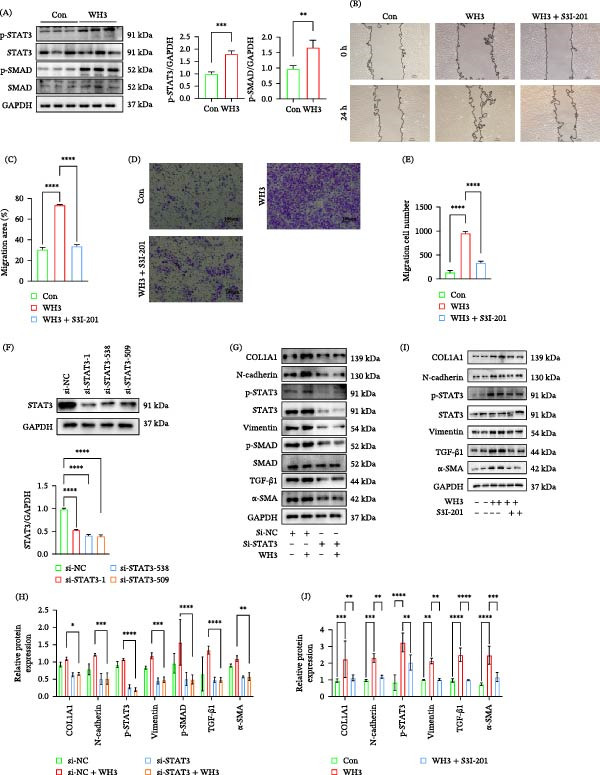

**Figure:**
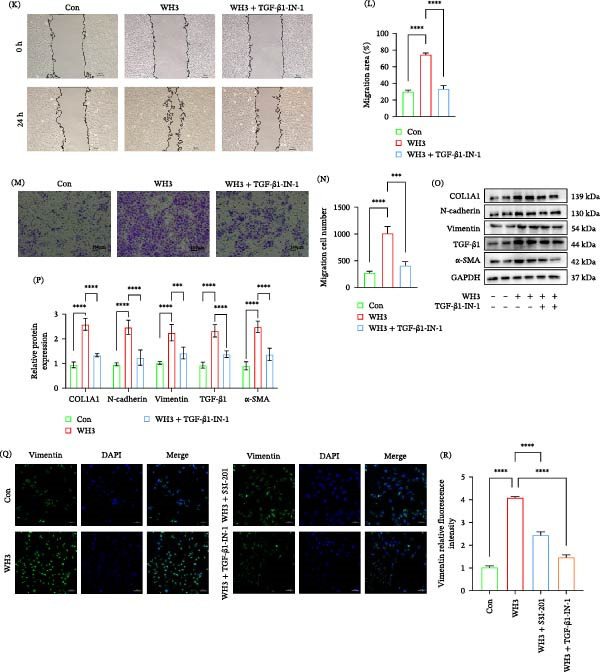

### 3.4. ROP16 Is an Essential Virulence Factor for *T. gondii*‐Induced EMT‐Like Changes in ARPE‐19

**Figure 4:**
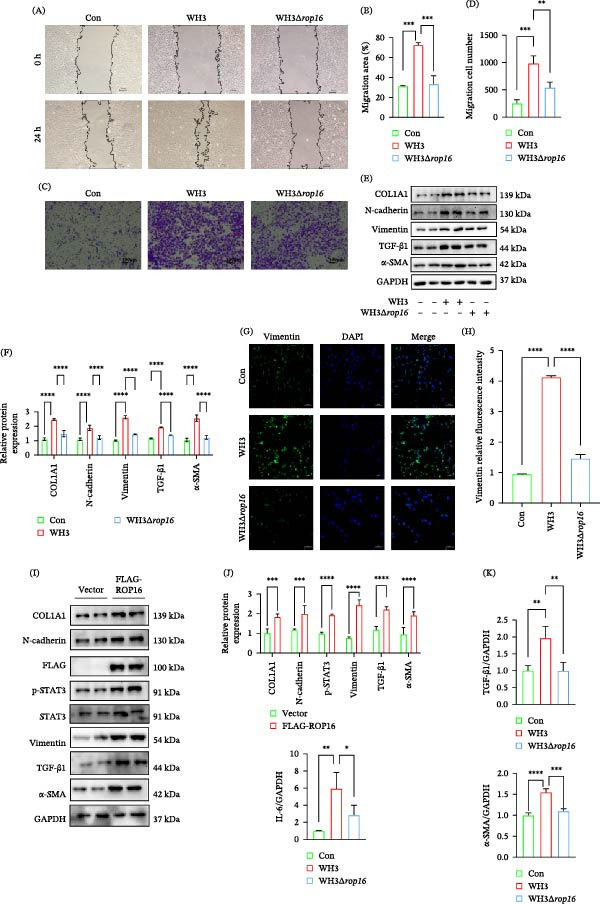
ROP16 is an essential virulence factor for *T. gondii*‐induced EMT‐like changes in ARPE‐19. (A, B) Representative images of ARPE‐19 cells infected with WH3 and WH3Δ*rop16* at 0 and 24 h after scratching. Scale bar: 100 μm. The relative scratch area was measured using ImageJ software (*n* = 3 per group). (C, D) Representative images of the transwell assay in ARPE‐19 cells with WH3 and WH3Δ*rop16* infection (*n* = 3 per group). The number of migrating cells was evaluated based on three random fields under a microscope, and quantitative analysis was performed using ImageJ software (*n* = 3 per group). Scale bar: 100 μm. (E, F) Protein expression and quantification of EMT‐related markers in ARPE‐19 cells with WH3 and WH3Δ*rop16* (*n* = 3 per group). (G, H) Representative confocal images of vimentin (green) in ARPE‐19 cells with WH3 or WH3Δ*rop16* infection. Quantitative analysis of vimentin fluorescence intensity (*n* = 3 per group). (I, J) Protein expression and quantification of EMT‐related markers in ARPE‐19 cells overexpressing with *rop16* (*n* = 3 per group). (K) qPCR detection of α‐SMA in ARPE‐19 cells with WH3 and WH3Δ*rop16* infection (*n* = 3 per group). Data are presented as mean ± SEM.  ^∗^ *p* < 0.05,  ^∗∗^ *p* < 0.01,  ^∗∗∗^ *p* < 0.001, and  ^∗∗∗∗^ *p* < 0.0001.

Our findings point to crucial roles of STAT3 and TGF‐β1 signaling in *T. gondii*‐induced EMT‐like changes. This process is likely mediated by parasite effectors such as ROP16, a known activator of STAT3 phosphorylation. To test the specific contribution of ROP16 to the initiation of EMT‐like changes, we infected ARPE‐19 cells with either wild‐type WH3 or a ROP16‐deletion mutant (WH3Δ*rop16*) and analyzed subsequent phenotypic changes related to EMT‐like changes. Wound‐healing assays revealed that WT infection significantly enhanced cell migration, whereas WH3Δ*rop16* infection markedly suppressed this effect (Figure 4A,B). Transwell assays further demonstrated that WT infection significantly increased the number of cells crossing the membrane, a phenotype reversed by WH3Δ*rop16* treatment (Figure 4C,D). Concurrently, molecular analysis revealed markedly reduced expression of EMT‐related markers in WH3Δ*rop16*‐infected cells compared to WT, suggesting that ROP16 deficiency suppresses WH3‐induced EMT‐like changes (Figure 4E,F). Compared to the WH3, WH3Δ*rop16* infection attenuated vimentin expression in ARPE‐19 cells, as confirmed by immunofluorescence (Figure 4G,H). To further validate ROP16 function, we overexpressed ROP16 in ARPE‐19 cells. Results showed that ROP16 overexpression sufficiently mimicked the *T. gondii* infection‐induced EMT‐like changes phenotype and caused upregulation of EMT‐related marker proteins (Figure 4I,J). Consistent with this phenotypic change, qPCR analysis further revealed that WH3 infection significantly upregulated profibrotic expression, while the WH3Δ*rop16* mutant impaired the transcription of α‐SMA (Figure 4K).

### 3.5. ROP16 Is an Essential Virulence Factor for *T. gondii*‐Induced Ocular EMT‐Like Changes In Vivo

**Figure 5:**
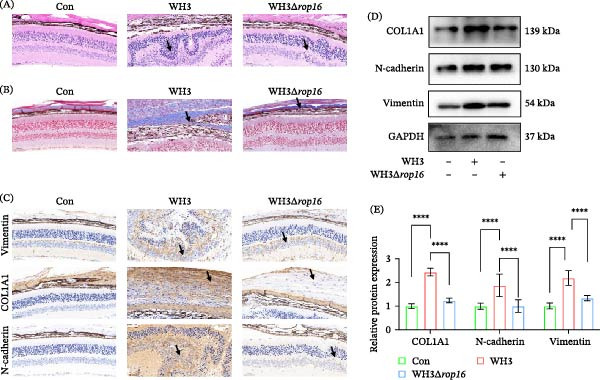
ROP16 is an essential virulence factor for *T. gondii*‐induced ocular EMT‐like changes in vivo. (A, B) Histopathological features of ocular tissues from C57BL/6 mice following intravitreal injection of PBS, WH3, and WH3Δ*rop16* strain, as shown by H&E and Masson staining. (C) Expression and distribution of vimentin, COL1A1, and N‐cadherin detected by immunohistochemical staining in ocular tissues from C57BL/6 mice intravitreally injected with PBS, WH3, and WH3Δ*rop16* strains (*n* = 5 per group). (D, E) Protein expression and quantitative analysis of EMT‐related markers in ocular tissues from C57BL/6 mice treated with WH3 and WH3Δ*rop16* strain. Data are presented as mean ± SEM. Arrows in subparts (A, B, and C) indicate areas of pathological damage, collagen deposition, or positively stained lesions.  ^∗^ *p* < 0.05,  ^∗∗^ *p* < 0.01,  ^∗∗∗^ *p* < 0.001, and  ^∗∗∗∗^ *p* < 0.0001.

In vivo, WH3Δ*rop16* infection exhibited markedly attenuated pathology compared to WH3. This was evidenced by reduced structural damage and collagen deposition on H&E and Masson staining (Figure 5A,B). Immunohistochemistry and Western blot analysis also confirmed the downregulation of the EMT‐related markers vimentin, COL1A1, and N‐cadherin in WH3Δ*rop16* infection, compared to WH3 group (Figure 5C–E). In summary, these findings demonstrate that ROP16 serves as a key effector protein regulating host EMT‐like changes during *Toxoplasma* infection, providing a mechanistic basis for its role in mediating cellular remodeling and pathological damage.

### 3.6. ROP16 Induces EMT‐Like Changes in ARPE‐19 via the STAT3 and TGF‐β1 Signaling Pathway

**Figure 6:**
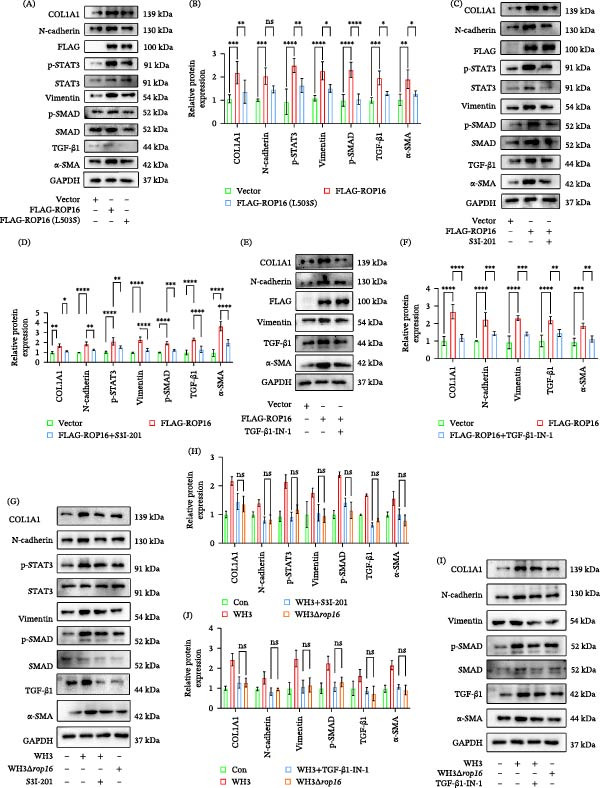
ROP16 induces EMT‐like changes in ARPE‐19 via the STAT3 and TGF‐β1 signaling pathways. (A, B) Western blot analysis and quantitative analysis of EMT‐related markers in ARPE‐19 cells under the following conditions: control, ROP16‐overexpressing, and ROP16 (L503S)‐overexpressing (*n* = 3 per group). (C, D) Western blot analysis and quantitative analysis of EMT‐related markers in control, ROP16‐overexpressing, and ROP16‐overexpressing cells treated with S3I‐201(*n* = 3 per group). (E, F) Western blot analysis of EMT‐related markers in control, ROP16‐overexpressing, and ROP16‐overexpressing cells treated with TGF‐β1‐IN‐1(*n* = 3 per group). (G, H) Western blot analysis and quantitative analysis of EMT markers in ARPE‐19 cells under different conditions: uninfected control, WH3 infection, WH3 infection + S3I‐201 treatment, and WH3Δ*rop16* infection (*n* = 3 per group). (I, J) Western blot analysis and quantitative analysis of EMT markers in ARPE‐19 cells under different conditions: uninfected control, WH3 infection, WH3 infection + TGF‐β1‐IN‐1 treatment, and WH3Δ*rop16* infection (*n* = 3 per group). Data are presented as mean ± SEM.  ^∗^ *p* < 0.05,  ^∗∗^ *p* < 0.01,  ^∗∗∗^ *p* < 0.001, and  ^∗∗∗∗^ *p* < 0.0001.

Previous studies have shown that ROP16, a kinase secreted by *T. gondii*, can phosphorylate multiple host proteins, including STAT3 during infection. To determine whether ROP16 mediates EMT‐like changes by phosphorylating STAT3, this study constructed a mutant at position 503 of ROP16, a site confirmed as its key active center for STAT3 phosphorylation. Subsequently, wild‐type and mutant ROP16 (L503S) were transfected into ARPE‐19 cells, and changes in the EMT‐like changes phenotype were assessed. The results revealed that the L503S mutation significantly impaired the ability of ROP16 to induce the expression of EMT markers, indicating that this process depends on its kinase activity and STAT3 phosphorylation (Figure 6A,B). To further validate the dependence of ROP16‐mediated EMT‐like changes on the STAT3 and TGF‐β1 pathway, we treated ARPE‐19 cells overexpressing ROP16 with STAT3 and TGF‐β1 inhibitors to compare the levels of EMT‐related proteins. The results demonstrated that upon blockade of the STAT3 and TGF‐β1 pathways, ROP16 no longer induced the upregulation of EMT‐related proteins (Figure 6C–F). Findings revealed that EMT marker expression levels in cells infected with WH3Δ*rop16* strain were comparable to those infected with WH3 parasites supplemented with STAT3 and TGF‐β1 inhibitors (Figure 6G–J). Collectively, these results indicate that ROP16 primarily promotes EMT‐like changes during *Toxoplasma* infection by activating the STAT3 and TGF‐β1 signaling pathway.

### 3.7. Blockade of the STAT3 and TGF‐β1 Signaling Pathway Can Alleviate OT

**Figure 7:**
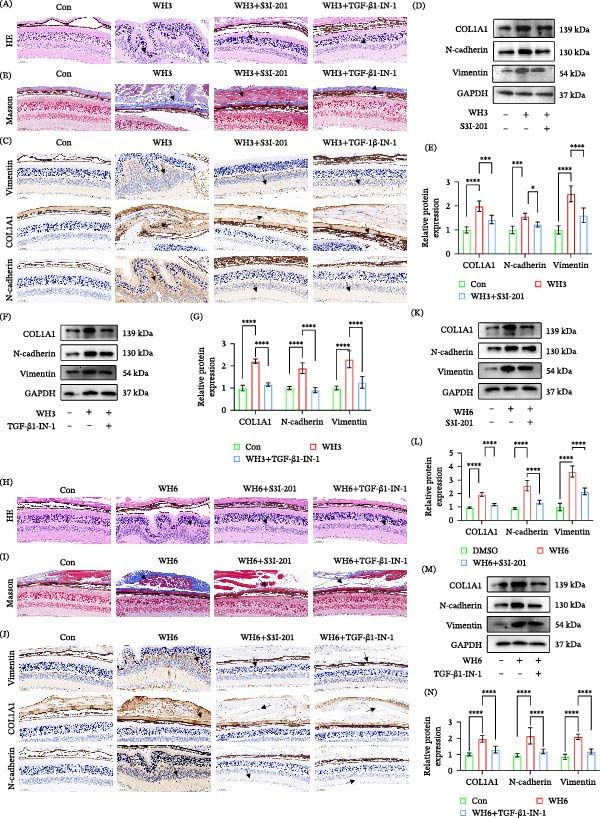
Blockade of the STAT3 and TGF‐β1 signaling pathway can alleviate ocular toxoplasmosis. (A, B) H&E and Masson staining demonstrating ocular tissue pathology in C57BL/6 mice from the following groups: control, WH3‐infected, WH3‐infected with S3I‐201 treatment, and WH3‐infected with TGF‐β1‐IN‐1 treatment. (C) Representative IHC images showing the expression profiles of vimentin, COL1A1, and N‐cadherin in ocular tissues across the treatment groups described in (A, B). (D, E) Protein expression and quantitative analysis of EMT‐related markers in ocular tissues from C57BL/6 mice from the following groups: control, WH3‐infected, WH3‐infected with S3I‐201 treatment (*n* = 5 per group). (F, G) Protein expression and quantitative analysis of EMT‐related markers in ocular tissues from C57BL/6 mice from the following groups: control, WH3‐infected, WH3‐infected with TGF‐β1‐IN‐1 (*n* = 5 per group). (H, I) H&E and Masson staining demonstrating ocular tissue pathology in C57BL/6 mice from the following groups: control, WH6‐infected, WH6‐infected with S3I‐201 treatment, and WH6‐infected with TGF‐β1‐IN‐1 treatment. (J) Representative IHC images showing the expression profiles of vimentin, COL1A1, and N‐cadherin in ocular tissues across the treatment groups described in (H, I). (K, L) Protein expression and quantitative analysis of EMT‐related markers in ocular tissues from C57BL/6 mice from the following groups: control, WH6‐infected, WH6‐infected with S3I‐201 treatment (*n* = 5 per group). (M, N) Protein expression and quantitative analysis of EMT‐related markers in ocular tissues from C57BL/6 mice from the following groups: control, WH6‐infected, WH6‐infected with TGF‐β1‐IN‐1 (*n* = 5 per group). Arrows in subparts (A, B, C, H, I, and J) indicate areas of pathological damage, collagen deposition, or positively stained lesions. Data are presented as mean ± SEM.  ^∗^ *p* < 0.05,  ^∗∗^ *p* < 0.01,  ^∗∗∗^ *p* < 0.001, and  ^∗∗∗∗^ *p* < 0.0001.

In vitro experiments have confirmed that STAT3 inhibitor (S3I‐201) and TGF‐β inhibitor (TGF‐β1‐IN‐1) can reverse the EMT‐like changes phenomenon induced by *T. gondii* infection in ARPE‐19 cells. To evaluate their therapeutic potential in vivo, we applied these two inhibitors in acute and chronic *T. gondii* ocular disease models in mice. In the acute infection model, HE staining showed that compared with the untreated infected control group, the ocular tissue pathological damage in infected mice treated with S3I‐201 or TGF‐β1‐IN‐1 was significantly reduced (Figure 7A). Masson staining further indicated that both inhibitors effectively inhibited the collagen deposition induced by WH3 infection (Figure 7B). Meanwhile, immunohistochemistry and Western blot analysis demonstrated that inhibitor treatment blocked the increase in COL1A1, N‐cadherin, and vimentin levels in infected ocular tissues (Figure 7C–G). These results suggest that inhibiting the STAT3 and TGF‐β1 pathway can significantly alleviate the ocular tissue damage and fibrotic response caused by WH3 infection. In the chronic infection model, we observed that S3I‐201 and TGF‐β1‐IN‐1 could also improve ocular pathological damage (Figure 7H), collagen deposition (Figure 7I), and significantly inhibit the expression of EMT‐related markers (such as N‐cadherin, vimentin, and COL1A1) (Figure 7J–N). Correspondingly, the mRNA levels of N‐cadherin and vimentin demonstrated a parallel trend (Supporting Information 2: Figure S1). In conclusion, this study demonstrates that STAT3 inhibitor S3I‐201 and TGF‐β1 inhibitor TGF‐β1‐IN‐1 can both exert protective effects on ocular tissues in acute and chronic *T. gondii* infections by inhibiting the EMT‐like changes process. This finding provides potential experimental evidence for targeted therapeutic strategies for *T. gondii* ocular disease.

## 4. Discussion

OT is an important cause of vision loss in patients with normal immune function, but its pathogenesis is not completely clear [36]. This study addresses a key gap by identifying EMT‐like changes as a crucial pathological process in OT. We have demonstrated that the *T. gondii* effector ROP16 drives EMT‐like changes through the STAT3 and TGF‐β1 signaling pathways. Importantly, drug inhibition of this pathway to reverse EMT‐like changes and alleviate ocular pathology reveals a new potential therapeutic strategy for this blinding condition.

EMT is a key process in the phenotypic transformation of cells, which is specifically manifested as epithelial cells losing their original polarity and intercellular adhesion, and acquiring the characteristics of interstitial cells. These newly acquired characteristics, including enhanced migration and invasion capabilities, as well as increased secretion of extracellular matrix (ECM), jointly promote the transformation of cells into fibroblast‐like morphology with motility [37, 38]. More and more evidence indicates that EMT plays a key role in the pathogenesis of various chronic diseases, including progressive kidney disease, pulmonary fibrosis, and liver fibrosis. In ocular diseases, EMT is also regarded as the core pathological mechanism of connection damage, inflammation, and fibrosis outcomes [26, 39]. The main clinical manifestation of OT is the formation of fibrotic scars. To explore whether *T. gondii* infection can induce ocular EMT‐like changes, this study conducted a systematic analysis using in vivo and in vitro infection models. A key clinical manifestation of OT is the formation of fibrotic scars. In the in vivo model, acute and chronic *T. gondii* infection mouse models were established. Histopathological examination revealed significant structural damage in the ocular tissues of both models, and Masson staining further indicated fibrotic changes, consistent with the clinical manifestations of OT. In in vitro experiments, using ARPE‐19, we observed that *T. gondii* infection significantly enhanced cell migration ability and led to a significant upregulation of EMT‐related marker proteins. In conclusion, this study provides evidence that *T. gondii* infection can induce EMT‐like changes in eye tissue. This discovery also provides new experimental insights into the fibrosis mechanism of toxoplasmosis, and puts forward potential treatment strategies for disease intervention through EMT‐like changes pathway.

Research on the pathogenesis of OT remains particularly limited. However, relevant studies have indicated that *T. gondii* infection can upregulate the phosphorylation level of STAT3 to regulate host biological activities. Previous reports have identified STAT3 as a key signaling molecule in fibrotic diseases, mediating fibrosis in the liver and kidneys [40, 41]. Our data demonstrated high expression of pSTAT3 upon *T. gondii* infection. Consequently, we inhibited STAT3 expression using specific inhibitors and siRNA, which effectively reversed the EMT‐like changes phenotype induced by *T. gondii* infection in vitro and in vivo. STAT3 is currently being actively investigated as a biomarker for a variety of diseases, particularly in cancer and immune‐related disorders. Examples include its role in tumor progression, inflammatory bowel disease, rheumatoid arthritis, and multiple sclerosis [42–44]. Our study demonstrates that STAT3 is significantly upregulated in ocular tissue following *T. gondii* infection. This finding raises the possibility of developing STAT3 as a biomarker for OT, potentially providing a noninvasive indicator that could enable earlier prediction of the progression toward fibrotic complications in OT compared to existing diagnostic methods. In both acute and chronic murine models of OT, pharmacological inhibition of STAT3 ameliorated pathological damage and attenuated fibrotic progression. These findings suggest a potential therapeutic strategy for treating OT. The role of STAT3 in EMT and fibrosis has been well documented in cancer and other inflammatory diseases. It often serves as a convergence point for multiple cytokine signals, such as IL‐6 [45]. Our study reveals that *T. gondii* hijacks this pathway, exploiting a core host signaling mechanism for its own benefit. This strategy may create a prosurvival and migratory environment for infected cells, potentially facilitating immune evasion and tissue colonization—albeit at the cost of activating destructive fibrotic pathways in the host.

*T. gondii* is a highly sophisticated protozoan pathogen that secretes various effector proteins to modulate host biological processes, thereby facilitating the establishment of persistent infection. Among these, ROP16 has been reported to phosphorylate host STAT3 via its residue at position 503, suggesting a potential role in inducing EMT‐like changes through STAT3 activation. The most significant contribution of this study is the identification of ROP16 as the key virulence factor responsible for this phenomenon. Using the WH3Δ*rop16* mutant strain, we demonstrated that the infection‐induced changes in EMT markers and functional alterations were substantially abolished compared to those elicited by the wild‐type strain. This effect was specifically attributable to the absence of ROP16, indicating that *T. gondii* promotes EMT‐like changes primarily through this effector molecule. These findings position ROP16 at the center of host–pathogen interactions that reprogram host cell identity. Our results are consistent with previous studies underscoring the pivotal role of ROP16 in regulating host gene expression, yet extend its functional scope to a direct involvement in driving pathological fibrosis via EMT‐like changes. We further elucidated the signaling mechanism downstream of ROP16 and identified the phosphorylation and nuclear translocation of STAT3 as a critical node. This was unequivocally demonstrated through both pharmacological inhibition and genetic silencing of STAT3, which effectively blocked the expression of EMT markers and abolished the enhanced cell migration induced by ROP16 from the WH3 strain. These findings confirm that STAT3 and TGF‐β1 are not merely associated with *T. gondii*‐induced EMT‐like changes, but are essential for ROP16‐mediated EMT‐like changes. By specifically modulating host cellular signaling, ROP16 may facilitate parasite dissemination by promoting migratory activity. Beyond inhibitors targeting the STAT3 and TGF‐β1 pathways as a therapeutic strategy, the development of neutralizing antibodies against ROP16 could provide a novel intervention approach for the treatment of OT.

Previous studies on OT have primarily focused on inflammatory responses and immunopathology, involving cytokines (CXCL9/10, IFN‑γ, and IL‑2) [46], immune cells (macrophages, CD8+ T cells, and dendritic cells) [47], disruption of the blood‑retinal barrier, and host genetic factors [48]. Building upon this foundation, the present study provides novel mechanistic evidence demonstrating, for the EMT‐like changes, that the *T. gondii* effector molecule ROP16 promotes EMT‐like changes in ocular tissue cells through activation of the STAT3 and TGF‑β1 pathways, as evidenced by the upregulation of vimentin, N‑cadherin, and COL1A1. Both previous and the current studies have observed elevated TGF‑β1 levels and involvement of the STAT3 pathway in the pathological process. However, previous studies generally did not examine EMT markers, nor did they directly link a specific parasite effector molecule to intraocular fibrosis. These similarities and differences suggest that the pathological mechanisms of OT involve a complex interplay between immune inflammation and host cellular phenotypic remodeling. Targeting the STAT3 and TGF‑β1 signaling pathways may hold translational potential for intervening in the progression of fibrosis in OT.

Despite the novel insights provided by this study, several limitations should be acknowledged. First, the experiments were primarily conducted in vitro using a human RPE cell line. The retinal microenvironment is inherently complex, involving multiple immune cells, cytokines, and intricate intercellular interactions. Therefore, the current findings require further validation using experimental models that more closely recapitulate physiological conditions. Second, EMT is a highly regulated process involving complex crosstalk and synergy among multiple signaling pathways. While our study focused on the STAT3 and TGF‐β1 pathways, other yet unidentified signaling mechanisms may also contribute to the regulation of EMT‐like changes in the context of OT, warranting further investigation.

Notwithstanding these limitations, this study provides important mechanistic insights into the role of the ROP16‐STAT3 and TGF‐β1 pathways in promoting EMT‐like changes in OT, highlighting STAT3 and TGF‐β1 as potential therapeutic targets. Future studies should validate these findings in animal models of OT. In particular, employing *T. gondii* strains carrying distinct ROP16 alleles (types I, II, and III) with varying STAT3 activation potentials would allow for the assessment of their differential impact on ocular EMT‐like changes. Such approaches may provide mechanistic explanations for the strain‐dependent differences in disease severity and fibrotic outcomes observed clinically, thereby advancing our understanding of disease pathogenesis and informing the development of targeted therapeutic strategies.

## 5. Conclusion

**Figure 8:**
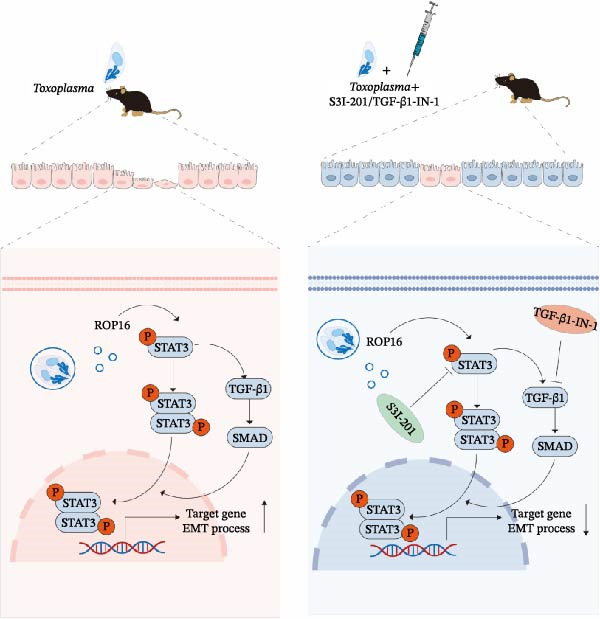
In the *T. gondii* infection model, the parasite‐secreted effector protein ROP16 promotes EMT‐like changes via the STAT3 and TGF‐β1 signaling pathways. Inhibition of this pathway inactivates both STAT3 and TGF‐β1 signaling, thereby suppressing EMT‐like changes and subsequent fibrosis.

In conclusion, our study delineates a novel molecular cascade in OT pathogenesis: the parasite effector ROP16 activates the host STAT3 and TGF‐β1 pathway to promote EMT‐like changes and subsequent fibrosis (Figure 8). This work provides a conceptual advance by linking a specific parasitic virulence mechanism to a fundamental pathological process in the eye. By identifying key actionable targets within this pathway, our findings pave the way for developing novel therapeutic paradigms aimed at mitigating the sight‐threatening fibrotic consequences of OT.

## Author Contributions

Li Yu and Lingling Song conceived the study. Lingling Song, Lihui Xu, and Yao Liu performed the experiments. Data collection and analysis were carried out by Yun Yang, Cong Wang, Ya Zhang, Qingli Luo, Yuanyuan Cao, and Yong Wang. All authors contributed to the interpretation of the results and manuscript revision.

## Funding

This work was funded by the National Natural Science Foundation of China (Grants 82072304 and 81871671) and Anhui Medical University Scientific Research Institution Construction and Promotion Plan Fund (Grant 2025xkjT012).

## Disclosure

All authors have approved the final manuscript.

## Ethics Statement

The Ethics Committee of the Institute of Health and Medicine, Hefei Comprehensive National Science Center (IHM‐AP‐2025‐162) approved this study.

## Conflicts of Interest

The authors declare no conflicts of interest.

## Supporting Information

Additional supporting information can be found online in the Supporting Information section.

## Supporting information


**Supporting Information 1** Table S1: Primers used in this study. Sense and antisense sequences are listed in the 5^′^→3^′^ orientation for primers targeting specific human and mouse genes, and siRNA sequences targeting STAT3 are provided.

**Supporting Information 2** Figure S1: qPCR analysis of EMT‐related marker expression in ocular tissues. qPCR analysis of EMT‐related marker expression in ocular tissues from C57BL/6 mice from the following groups: control, WH6‐infected, WH6‐infected with S3I‐201 treatment, and WH6‐infected with TGF‐β1‐IN‐1 treatment. Data are presented as mean ± SEM.  ^∗^ *p* < 0.05,  ^∗∗^ *p* < 0.01,  ^∗∗∗^ *p* < 0.001, and  ^∗∗∗∗^ *p* < 0.0001.

## Data Availability

The data that support the findings of this study are available from the corresponding author upon reasonable request.
