# Supplementary material for: ROP16 Promotes Epithelial‐Mesenchymal Transition‐Like Changes in Ocular Toxoplasmosis via STAT3 and TGF‐β1 Pathways
Source: Transbound Emerg Dis. 2026 May 22;2026:9126072. doi: 10.1155/tbed/9126072 (PMC13197621; doi:10.1155/tbed/9126072)
Supplement: Supplementary file 1 — Supporting Information 1 Table S1: Primers used in this study. Sense and antisense sequences are listed in the 5′→3′ orientation for primers targeting specific human and mouse genes, and siRNA sequences targeting STAT3 are provided. [file TBED-2026-9126072-s001.docx]

| Primer name | Sense (5′–3′) | Antisense (5′–3′) |
| --- | --- | --- |
| IL-6(Human) | AGTGAGGAACAAGCCAGAGC | TGGCATTGCATCCCTGAGTT |
| GAPDH(Human) | GTCAAGGCTGAGAACGGGAA | AAATGAGCCCCAGCCTTCTC |
| TGF-β1(Human) | GCAACAATTCCTGGCGATACCTC | CCTCCACGGCTCAACCACTG |
| α-SMA(Human) | CAATGTCCTATCAGGGGGCAC | CGGCTTCATCGTATTCCTGTT |
| IL-1β (Human) | TCCAGGGACAGGATATGGAG | TCTTTCAACACGCAGGACAG |
| GAPDH(mouse) | TGATGGGTGTGAACCACGAG | AGTGATGGCATGGACTGTGG |
| Vimentin(mouse ) | TGCTTCAAGACTCGGTGGAC | GGTCATTCAGCTCCTGCAGT |
| N-cadherin(mouse) | CACTGCCATTGATGCGGATG | TGCCACAGTGATGATGTCCC |
| SiSTAT3-1 | GGCCCAAUGGAAUCAGCUA | UAGCUGAUUCCAUUGGGCC |
| Si STAT3-509 | GACUUUGAUUUCAACUAUA | UAUAGUUGAAAUCAAAGUC |
| Si STAT3-538 | GAGUCAAGGAGACAUGCAA | UUGCAUGUCUCCUUGACUC |

*Supplementary Table S1:* Primers used in this study.
