# Supplementary figures and images for: ROP16 Promotes Epithelial‐Mesenchymal Transition‐Like Changes in Ocular Toxoplasmosis via STAT3 and TGF‐β1 Pathways
Source: Transbound Emerg Dis. 2026 May 22;2026:9126072. doi: 10.1155/tbed/9126072 (PMC13197621; doi:10.1155/tbed/9126072)

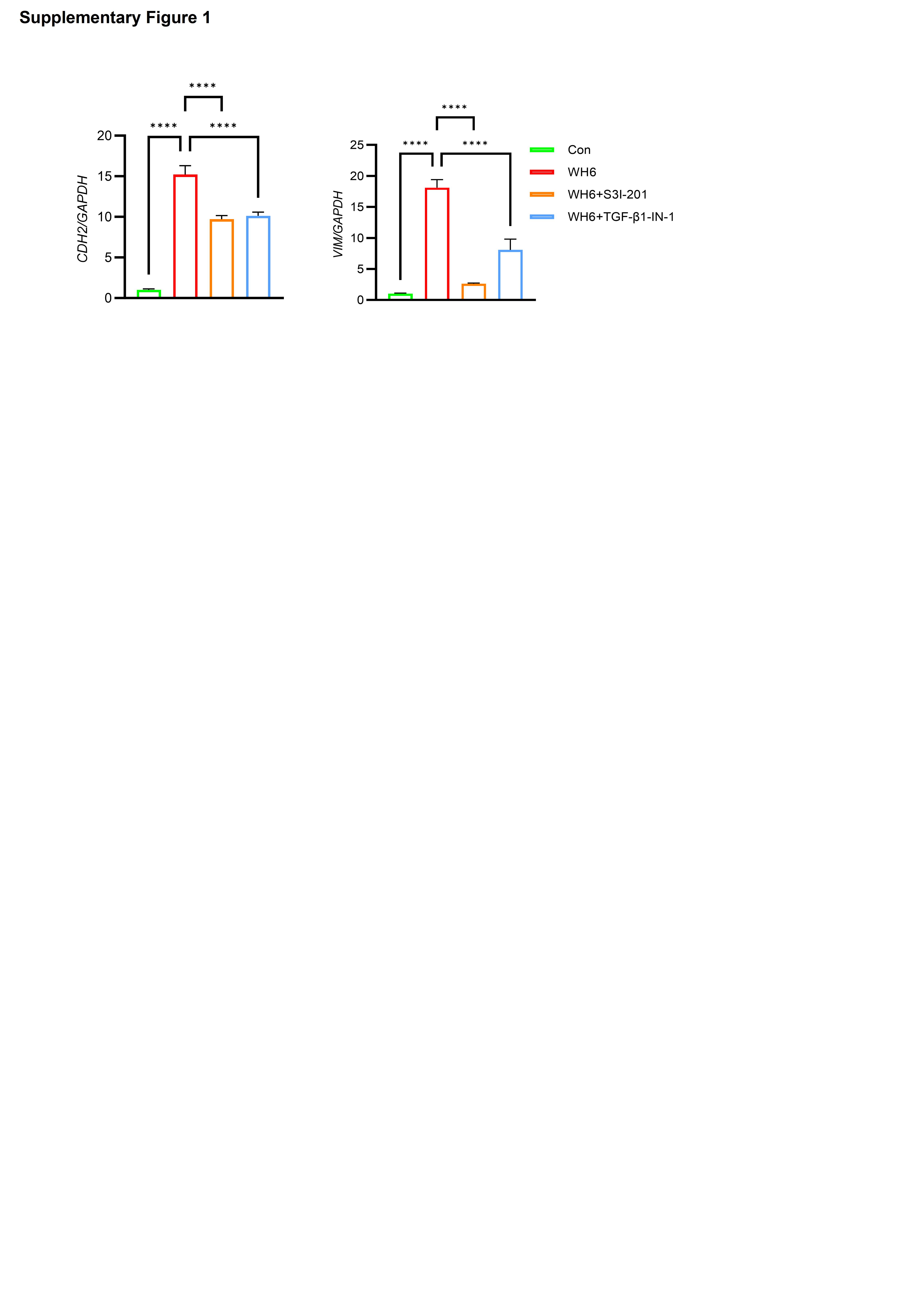

Supplement: Supplementary file 2 — Supporting Information 2 Figure S1: qPCR analysis of EMT‐related marker expression in ocular tissues. qPCR analysis of EMT‐related marker expression in ocular tissues from C57BL/6 mice from the following groups: control, WH6‐infected, WH6‐infected with S3I‐201 treatment, and WH6‐infected with TGF‐β1‐IN‐1 treatment. Data are presented as mean ± SEM. ∗ p < 0.05, ∗∗ p < 0.01, ∗∗∗ p < 0.001, and ∗∗∗∗ p < 0.0001. [file TBED-2026-9126072-s002.jpg]
